# Postoperative Morbidity and Failure to Rescue in Surgery for Gastric Cancer: A Single Center Retrospective Cohort Study of 1107 Patients from 1972 to 2014

**DOI:** 10.3390/cancers12071953

**Published:** 2020-07-18

**Authors:** Christian Galata, Ulrich Ronellenfitsch, Susanne Blank, Christoph Reißfelder, Julia Hardt

**Affiliations:** 1 Department of Surgery, Universitätsmedizin Mannheim, Medical Faculty Mannheim, Heidelberg University, D-68167 Mannheim, Germany; christian.galata@umm.de (C.G.); susanne.blank@umm.de (S.B.); julia.hardt@umm.de (J.H.); 2 Department of Visceral, Vascular and Endocrine Surgery, University Hospital Halle (Saale), Martin-Luther-University Halle-Wittenberg, D-06120 Halle (Saale), Germany; ulrich.ronellenfitsch@uk-halle.de

**Keywords:** gastric cancer, gastrectomy, complications, mortality, failure to rescue

## Abstract

Background: The aim of this study was to evaluate postoperative morbidity, mortality, and failure to rescue following complications after radical resection for gastric cancer. Methods: A retrospective analysis of the surgical database of patients with gastroesophageal malignancies at our institution was performed. All consecutive patients undergoing R0 gastrectomy for pT1–4 M0 gastric adenocarcinoma between October 1972 and February 2014 were eligible for this analysis. Patients were divided into two groups according to the date of surgery: an early cohort operated on from 1972–1992 and a late cohort operated on from 1993–2014. Both groups were compared regarding patient characteristics and surgical outcomes. Results: A total of 1107 patients were included. Postoperative mortality was more than twice as high in patients operated on from 1972–1992 compared to patients operated on from 1993–2014 (6.8% vs. 3.2%, *p* = 0.017). Between both groups, no significant difference in failure to rescue after major surgical complications was observed (20.8% vs. 20.5%, *p* = 1.000). Failure to rescue after other surgical and non-surgical complications was 37.8% in the early cohort compared to 3.2% in the late cohort (*p* < 0.001). Non-surgical complications accounted for 71.2% of lethal complications between 1972 and 1992, but only for 18.2% of lethal complications between 1993 and 2014 (*p* = 0.002). Conclusion: In the course of four decades, postoperative mortality after radical resection for gastric cancer has more than halved. In this cohort, the reason for this decrease was reduced mortality due to non-surgical complications. Major surgical morbidity after gastrectomy remains challenging.

## 1. Introduction

Resection for gastric cancer is regarded as a high-risk surgery with significant morbidity and mortality [1,2]. Failure to rescue, defined as the mortality among patients with postoperative complications, has recently been of interest in this context. Patients with gastric cancer have been reported to have even higher failure to rescue rates than patients undergoing esophageal resections [3]. In recent years, the focus of large-scale multicenter studies has been to examine variations in surgical outcomes among patients undergoing surgery for gastric cancer between different institutions [4,5]. Current evidence suggests that high-volume surgical centers might have lower mortality rates [6,7]. A significant reduction in postoperative mortality and failure to rescue from 2011 to 2014 has been reported in a recent study from the Netherlands, where the authors concluded that these effects were at least in part due to increased centralization and newly introduced minimum volume requirements [3]. In 2019, a register-based study from Germany evaluated postoperative mortality in non-bariatric gastric resections among patients operated on between 2010 and 2015 [8]. All these studies compared surgical outcomes of high-volume and low-volume centers over short periods of time. However, changes in surgical outcomes over longer periods of time are rarely reported. Developments in surgical techniques and perioperative management over recent decades are likely to impact patient outcomes. However, relevant results are very often not available due to a lack of long-term data. Therefore, the rationale for this study was to evaluate patients undergoing radical resection for gastric cancer from 1972 to 2014 regarding changes in postoperative morbidity, mortality, and failure to rescue. The same patient collective has recently been investigated regarding the impact of postoperative complications on overall survival. The results showed that postoperative complications are a significant risk factor for poor overall survival, an effect which was mainly caused by complication-associated early mortality [9]. A further objective of the present study was to examine whether—as expected in view of the medical progress—postoperative complications and failure to rescue after gastric cancer surgery were reduced over the course of the four decades studied. In order to examine this hypothesis in more detail, the total study time period was divided in two equally long periods (early versus late) and the outcomes of the two periods were compared.

## 2. Results

### 2.1. Patients’ Characteristics

Between October 1972 and February 2014, a total of 1107 consecutive patients underwent R0 resection for pT1–4 M0 gastric adenocarcinoma at our institution and were included in the analysis. The standard approach for these patients at our institution was open total or subtotal (4/5) gastrectomy with D2 lymphadenectomy (LAD). To investigate changes in postoperative morbidity and mortality, patients were stratified into two cohorts according to the date of operation, 1972–1992 and 1993–2014. During the early period, 761 patients underwent surgery as compared to 346 patients during the late period.

Patients’ characteristics are shown in Table 1. In both cohorts, there were more males than females, 55.1% and 54.6%, respectively. Median patient age was significantly higher in the 1993–2014 group (67 vs. 65 years, *p* < 0.001). After the year 1992, total gastrectomy was more frequently performed than subtotal gastrectomy (56.6% vs. 42.7%, *p* < 0.001). The rate of multivisceral resections was not significantly different (47.0% in the late cohort vs. 45.3% in the early cohort, *p* = 0.645). However, splenectomies were performed significantly more often from 1972 to 1992 (40.2% vs. 20.5%, *p* < 0.001), whereas cholecystectomies were more commonly performed from 1993 to 2014 (23.5% vs. 5.4%, *p* < 0.001).

### 2.2. Tumor Characteristics

Tumor characteristics are shown in Table 2. Tumor characteristics were comparable between both periods, with no significant differences in tumor location (*p* = 0.163), pT (*p* = 0.359), pN (*p* = 0.119), or American Joint Committee on Cancer/Union Internationale Contre le Cancer (AJCC/UICC) stages (*p* = 0.343) observed. Only for the histological classification based on Laurén’s criteria were significantly more cancers of the diffuse type observed in the later years (*p* < 0.001).

### 2.3. Postoperative Outcomes

Postoperative outcomes are shown in Table 3. The median length of hospital stay was significantly shorter in the period from 1993 to 2014 compared to the period from 1972 to 1992 (*p* < 0.001). The overall postoperative complication rate was higher in the later cohort (31.9% vs. 22.3%, *p* = 0.001). However, when the rate of major surgical complications (defined as anastomotic leak, postoperative abdominal abscess, fascial dehiscence, peritonitis, sepsis, secondary hemorrhage, or relaparotomy for any reason) was investigated, no statistically significant differences were observed between both groups (*p* = 0.071). Similarly, the anastomotic leak (including duodenal stump leaks) rates did not differ significantly (6.3% in the late cohort vs. 3.8% in the early cohort, *p* = 0.084).

Both 30-day mortality (6.0% vs. 1.7%) and in-hospital mortality (6.8% vs. 3.2%) were significantly higher in the 1972 to 1992 cohort as compared to the 1993 to 2014 cohort. No statistically significant difference in the failure to rescue rate after major surgical complications was observed between both time periods (20.5% in the late cohort vs. 20.8% in the early cohort, *p* = 1.000). In contrast, failure to rescue after non-major surgical complications and non-surgical complications was significantly more common from 1972 to 1992 as compared to the period from 1993 to 2014 (37.8% vs. 3.2%, *p* < 0.001). Mortality-associated complications are shown in Table 4. In the early cohort, non-surgical complications accounted for 71.2% of all lethal complications as compared to 18.2% in the late cohort, whereas major surgical morbidity accounted for 28.8% of complication-related deaths from 1972 to 1992 as compared to 81.8% from 1993 to 2014 (*p* = 0.002). For patients who underwent splenectomy (Table 5), a higher rate of major surgical (*p* = 0.016) and overall complications (*p* = 0.005) was observed in the late cohort (major morbidity 22.7%, overall morbidity 40.9%) compared to the early cohort (major morbidity 11.1%, overall morbidity 23.5%). However, patients who underwent splenectomy in the late cohort had significantly higher T-stages (*p* = 0.049), higher N-stages (*p* < 0.001), and higher UICC stages (*p* = 0.001).

### 2.4. Risk Factors for Postoperative Mortality

Univariable and multivariable analyses were performed to identify potential risk factors for postoperative mortality. In the entire cohort (1972–2014), patient age and the occurrence of overall postoperative complications, major surgical complications, and anastomotic leak were factors significantly associated with postoperative death in univariable analysis (Table 6). When multivariable analysis was performed, major surgical complications, anastomotic leak, and older patient age remained in the statistical model as factors associated with postoperative in-hospital mortality (Table 7). Subgroup analyses of patients operated on from 1972 to 1992 and from 1993 to 2014 returned similar results (Table 6 and Table 7).

## 3. Discussion

This study presents one of the rare reports on the results of surgery for gastric cancer in a large cohort of 1107 consecutive patients (1972 to 1992: *n* = 761 and 1993 to 2014: *n* = 346), over a time period of more than four decades at a European university hospital.

The main finding of the study was that 30-day and in-hospital mortality were significantly lower in the later period. Major surgical complications, anastomotic leak, and older patient age were independent risk factors for postoperative in-hospital mortality in multivariable analysis. However, it is not unlikely that age would have dropped out as a risk factor for postoperative mortality if other known risk factors, such as serum albumin level, Eastern Cooperative Oncology Group (ECOG) performance status, and American Society of Anesthesiologists (ASA) physical status, which were not available in our data set, had been included in the analysis.

Analysis of mortality-associated complications revealed that non-surgical complications accounted for the large majority (71.2%) of all lethal complications in patients with surgery between 1972 and 1992, as compared to only 18.2% of lethal complications in patients with surgery between 1993 and 2014 (*p* = 0.002). Correspondingly, failure to rescue rates for major surgical complications did not differ between the two time periods, while failure to rescue occurred significantly more frequently among patients with non-major surgical and non-surgical complications in the cohort operated on between 1972 and 1992 (37.8% vs. 3.2%). This retrospective study cannot provide further details on the reasons for this shift regarding the type of complications associated with a lethal course. The main driver is most likely the fact that the treatment of acute medical complications, such as pulmonary embolism or myocardial infarction that frequently led to death in former times, has tremendously improved over recent decades.

An analysis of the register data of the Dutch Upper GI Cancer Audit showed similar rates: postoperative mortality after gastric cancer surgery ranged from 7.7% in 2011 to 3.8% in 2014 and failure to rescue occurred in 38.0% of patients with a complication in 2011 and in 19.0% in 2014 [3]. The authors attribute this to quality improvement measures, such as multidisciplinary therapy standards and minimum surgical volume requirements. A recently published large observational study from Germany, using national hospital discharge data (72,528 cases of non-bariatric gastric surgery), revealed that the prevention of mortality after complex gastric surgery mainly depends on the ability to rescue patients with complications. Failure to rescue rates were 28.1% in very low-volume hospitals versus 22.7% in very high-volume hospitals [8]. According to most definitions, institutions with an average of about 50 gastroesophageal resections for malignant indications per year are regarded as high-volume centers [10]. The reported mortality rates of our patients operated on between 1972 and 1992 were consistent with the findings of a recent retrospective cohort study based on American College of Surgeons National Surgery Quality Improvement Program (ACS NSQIP) data: 30-day mortality was 5.2% [11]. This also compares well to the results from a large US cohort study analyzing the National Inpatient Sample (NIS) data of 13,354 patients undergoing gastric resection for malignancy with an in-hospital mortality rate of 6.0% [12]. A much lower short-term mortality was reported by the authors of a Cochrane systematic review investigating the outcomes of laparoscopic versus open gastrectomy for gastric cancer; the pooled short-term mortality of eleven randomized controlled trials (RCTs) was only 0.4% (11/2635) [13].

Further comparison of the two time periods revealed a few more statistically significant differences. Not surprisingly, the length of hospital stay has decreased in the more recent decades, which is most likely the result of the implementation of a modern and evidence-based clinical pathway for gastric resections that follows the principles of the enhanced recovery after surgery (ERAS^®^) and “fast track” concepts. Moreover, significantly older patients were operated on, which speaks for the advances of modern perioperative medicine that has made it possible to offer curative surgery even to elderly patients. The fact that the number of cases in the early time period was more than twice as high as in the later time period (761 vs. 346) reflects the decline in the incidence of “true” gastric cancer and a shift towards more proximal tumor locations, such as the cardia and distal esophagus, in Europe during recent decades [14]. In general, the clinicopathologic features of the patients in our analysis were consistent with other long-term analyses of patients with surgery for gastric cancer [15].

Interestingly, there were more overall complications (31.9% vs. 22.3%, *p* = 0.001) in the later time period (1993–2014), which may have multifactorial etiology. First, in the later time period, there were significantly more total gastrectomies, which are known to be associated with a higher risk for postoperative morbidity than subtotal gastrectomies. The most plausible explanation for the increase in total gastrectomies is the significantly higher number of cancers of the diffuse type according to Laurén’s criteria in the late cohort. Furthermore, the fact that significantly older, and thus potentially frailer and more comorbid patients, were operated on in the 1990s and 2000s could also have resulted in a higher overall complication rate. Besides, the latter may partly explain why we did not find a decrease in major surgical morbidity despite the far-reaching technical and medical advances that took place during the four decades included in our analysis. Nevertheless, the actual clinical relevance of the age difference between the two cohorts remains to be discussed. Life expectancy has also increased during the observation period of more than four decades, and so it may be that the median age difference of 2 years is clinically irrelevant, because the older patients of the late cohort were not biologically older than the chronologically younger patients of the early cohort. We did not evaluate the impact of the extent of LAD on postoperative morbidity and mortality, as the extent of LAD was not thoroughly documented in the early decades of the database. However, the trend towards a more radical LAD (D2 or D3 vs. D1) over time may have contributed to an increase in the overall complication rate in our later cohort, which has been reported in several RCTs from Western countries [16,17]. In this regard, one could also argue that, despite more radical operations (more total gastrectomies and more extensive LAD), a rise in major complications did not occur, which could be the result of advances in surgical techniques and perioperative management. Neoadjuvant treatment of gastric cancer patients was only introduced in the years 2005 to 2006 and thus administered only to the absolute minority of patients included in our study. Therefore, we chose not to include this parameter in our analyses. Yet, most studies on this found no increase in postoperative morbidity or mortality after neoadjuvant treatment [18,19].

There are some limitations to our study. First, the retrospective nature and the inherent potential for misclassification may limit the validity of our data. Second, there may be confounding variables not recorded in our database. Therefore, we cannot exclude the possibility that factors not tracked in the database may have contributed to our findings. Third, missing data and changes in coding and classifications over such a long period as forty-two years may limit the validity of our results. Fourth, the exclusion of R1 resections, because of the aim to exclusively investigate patients in a curative situation and to generate a study population as homogeneous as possible regarding their risk profile for complications, may be called into question in the context of analyzing short-term outcomes after gastric cancer surgery. Thus, the exclusion of R1 resections may be interpreted as a potential limitation of our study because it could have biased the results. Fifth, the use of the Clavien Dindo Classification would have been desirable to increase the comparability of the results. However, the Clavien Dindo Classification was first described in 2004, whereas our institutional database collected cases from 1972 to 2014. Thus, in most cases, complications were not documented according to the Clavien Dindo Classification and a retrospective classification would have been error-prone and unreasonable. Sixth, data on the extent of lymphadenectomy and 90-day mortality would have been of interest in the context of the present study but were not available in sufficient continuity and quality over the long observation period for inclusion into the analyses. The strengths of this study lie in its large sample size, the consecutive, homogenous cohort (only gastric adenocarcinomas, only patients operated on with curative intent, one center), and the long time period covered. Most cohort studies based on data from registers or institutional databases which investigated similar research study objectives included rather heterogeneous patient populations, sometimes even patients who underwent gastric surgery for benign disease. With the aim to reduce bias and confounding factors due to heterogeneity among the included cases, we defined very precise inclusion and exclusion criteria. Nevertheless, the included patient cohort and the setting are still representative in the context of the investigated study objective. Therefore, we assume that the study results have a high external validity and generalizability.

## 4. Materials and Methods

### 4.1. Ethics Approval

Ethics board approval was obtained from the Medical Ethics Commission II of the Medical Faculty Mannheim, Heidelberg University, Mannheim, Germany (2019-849R). The study was performed according to the Declaration of Helsinki.

### 4.2. Study Design

The present study is a single-center retrospective cohort study using data from a prospectively run institutional database.

### 4.3. Setting and Participants

Medical records from 2252 consecutive patients operated on at the Department of Surgery, University Medical Centre Mannheim, Heidelberg University, Mannheim, Germany between October 1972 and February 2014 were examined, and patients with pT1–4 M0 adenocarcinomas of the stomach who underwent R0 resections were identified. Patients with Barrett’s carcinoma and gastric remnant cancer were excluded, as were patients with atypical gastric or esophageal resections. Tumors of the subcardial stomach (Siewert type III) were included, whereas esophagogastric junctional adenocarcinomas (Siewert types I and II) were excluded, as these are classified and staged according to the esophageal scheme in the current AJCC/UICC staging system. Finally, 1107 of the 2252 patients met the inclusion criteria and could be further analyzed. For the present analyses, no follow-up of patients was required.

### 4.4. Variables, Data Sources, and Risk of Bias

Data on patient, procedure, and tumor characteristics were taken from the institutional database for gastroesophageal malignancies. Patient and procedure characteristics included gender, age, type of gastrectomy (total versus subtotal), and the extent of resection in the case of multivisceral resection. Tumor characteristics comprised tumor location (non-antropyloric versus antropyloric), Laurén’s classification, histological type, and pT and pN categories, as well as the AJCC/UICC stages. The latter were available according to the 5th edition for all gastric cancers operated on between 1972 and 2001. The 6th and 7th editions of the AJCC/UICC classification were used on cases from 2002 until 2009 and from 2010 until 2014, respectively. Before analysis, all patients included in this study were restaged according to the 6th edition of the AJCC/UICC staging system for gastric cancer, which was the most recent edition based on which the restaging of all patients was possible, in order to achieve a uniform classification. Data on surgical and non-surgical complications were extracted from the database. Major surgical complications were defined as the documentation of at least one of the following events during the postoperative course: anastomotic leak (including duodenal stump leak), postoperative (abdominal) abscess, fascial dehiscence, peritonitis, sepsis, secondary hemorrhage, and relaparotomy for any reason. When multiple complications occurred, the most severe complication was recorded in the database. In cases where multiple complications were recorded, the most severe complication was used in this analysis. Complication-related postoperative mortality was recorded and presented as early postoperative (30-day) and general in-hospital mortality. Failure to rescue, defined as the mortality among patients with postoperative complications, was calculated as the ratio of the number of patients who died due to certain complications (numerator) to the number of all patients who suffered these complications (denominator).

The options to minimize the risk for bias were limited due to the retrospective nature of the study. As with any other retrospective database review, bias cannot be excluded from the present study. Nevertheless, by using strict inclusion and exclusion criteria, we tried to obtain a study cohort that was as homogeneous as possible, thus minimizing the risk of bias and confounding. Moreover, the data collection was the same in both study periods.

### 4.5. Statistical Analysis

The median, together with the interquartile range (IQR), was presented for skewed variables. Qualitative variables were quoted as absolute and relative frequencies. Quantitative variables, like age, hospital stay, and number of postoperative complications, were treated as such in the statistical analyses. The variable “date of surgery” was dichotomized into an early (1972–1992) and a late (1993–2014) group. The Mann–Whitney U test was used to compare continuous variables that were not normally distributed. For dichotomous variables, Fisher’s exact test was used. The Cochran–Armitage test for trend was used to assess associations between a dichotomous variable and an ordinal variable with more than two categories. All statistical tests for the comparison of two groups were two-tailed. A test result was considered statistically significant if *p* < 0.05. For the binary outcome “postoperative mortality”, a multiple logistic regression analysis was done. Odds ratios are presented together with their 95% confidence intervals (CI). Variables that were statistically significant in the univariable analyses were entered in the multivariable analysis. In the multivariable analysis, a backward stepwise selection based on the probability of the Wald statistic was used and a significance level of α = 0.10 was chosen to detect several parameters that might have influenced the outcome. Statistical analyses were performed using IBM SPSS Statistics (version 25, IBM Corp., Armonk, NY, USA).

## 5. Conclusions

In conclusion, major surgical morbidity after gastrectomy remains a major challenge despite advances in perioperative care and surgical techniques. Over the course of four decades, postoperative mortality after radical resection for gastric cancer has more than halved, which was mainly caused by a reduction in mortality-associated non-surgical complications and an improved ability to rescue patients with complications. Therefore, alertness and adequate infrastructure in order to avoid failure to rescue are paramount for achieving acceptable outcomes after gastrectomy.

## Figures and Tables

**Table 1:** Patient and procedure characteristics.

Parameter	1972–1992 (*n* = 761)		1993–2014 (*n* = 346)		*p*
	*n* or median	% or IQR	*n* or median	% or IQR	
Gender					0.897
Female	342	44.9	157	45.4	
Male	419	55.1	189	54.6	
Age	65	56–71	67	57–74	**0.001**
Gastrectomy					**<0.001**
Total	325	42.7	196	56.6	
Subtotal	436	57.3	150	43.4	
Multivisceral resection	345	45.3	158	47.0	0.645
Splenectomy	306	40.2	69	20.5	**<0.001**
Cholecystectomy	41	5.4	79	23.5	**<0.001**
Intestinal resection	17	2.2	12	3.6	0.222
Hepatic procedure	9	1.2	11	3.3	**0.025**
Pancreatic procedure	21	2.8	8	2.4	0.840
Missing data	0		10		

*p* values in bold type indicate statistical significance.

**Table 2:** Tumor characteristics.

Parameter	1972–1992 (*n* = 761)		1993–2014 (*n* = 346)		*p*
	*n*	%	*n*	%	
Tumor location					0.163
Non-antropyloric	474	62.3	200	57.8	
Antropyloric	287	37.7	146	42.2	
Laurén classification					**0.001**
Non-diffuse	473	62.2	165	51.6	
Diffuse	287	37.8	155	48.4	
Missing data	1		26		
pT category					0.359
T1–T2	588	77.3	258	74.6	
T3–T4	173	22.7	88	25.4	
pN category					0.119
pN0	377	49.5	153	44.3	
pN1–3	384	50.5	192	55.7	
Missing data	0		1		
AJCC/UICC stage					0.343
IA–IB	358	47.0	157	45.5	
II	183	24.0	80	23.2	
IIIA–IIIB	180	23.7	83	24.1	
IV	40	5.3	25	7.2	
Missing data	0		1		

*p* values in bold type indicate statistical significance. AJCC: American Joint Committee on Cancer; UICC: Union Internationale Contre le Cancer.

**Table 3:** Postoperative outcomes.

Outcome	1972–1992 (*n* = 761)		1993–2014 (*n* = 346)		*p*
	*n*	% or IQR	*n*	% or IQR	
Postoperative morbidity					
Overall complications	170	22.3	107	31.9	**0.001**
Major surgical complications	72	9.5	44	13.1	0.071
Anastomotic leak	29	3.8	21	6.3	0.084
Other surgical/non-surgical complications	98	12.9	63	18.8	**0.012**
Missing data	0		11		
Postoperative mortality					
30-day mortality	46	6.0	6	1.7	**0.001**
In-hospital mortality	52	6.8	11	3.2	**0.017**
Missing data	0		2		
Failure to rescue					
Overall complications	52/170 *	30.6	11/107 *	10.3	**<0.001**
Major surgical complications	15/72 *	20.8	9/44 *	20.5	1.000
All other complications	37/98 *	37.8	2/63 *	3.2	**<0.001**
Hospital stay (days)	15	13–19	14	11–18	**<0.001**
Missing data	3		3		

* The denominator indicates the total number of patients with each respective complication. *p* values in bold type indicate statistical significance.

**Table 4:** Mortality-associated complications.

Type of Lethal Complication	1972–1992 Total Cases: *n* = 761 Postoperative Mortality: *n* = 52		1993–2014 Total Cases: *n* = 346 Postoperative Mortality: *n* = 11	
	n	% of mortality	n	% of mortality
Major surgical complications	15	28.8	9	81.8
Anastomotic leak	10	19.2	8	72.7
Relaparotomy(other reason/not specified)	4	7.7	0	0
General sepsis	1	1.9	1	9.1
Non-surgical complications	37	71.2	2	18.2
Cardiovascular	13	25.0	0	0
Cardiorespiratory	11	21.2	1	9.1
Respiratory	11	21.2	0	0
Other	2	3.8	1	9.1

**Table 5:** Patients with splenectomy.

Variable	1972–1992 (*n* = 306)		1993–2014 (*n* = 69)		*p*
	*n*	%	*n*	%	
Overall complications	34	11.1	15	22.7	****0.005****
Major surgical complications	72	23.5	27	40.9	****0.016****
Anastomotic leak	17	5.6	6	9.1	0.267
Missing data	0		3		

*p* values in bold type indicate statistical significance.

**Table 6:** Variables associated with postoperative mortality in univariable analysis.

Variable	Overall Cohort (1972–2014)		*p*	Early Group (1972–1992)		*p*	Late Group (1993–2014)		*p*
	OR	95% CI		OR	95% CI		OR	95% CI	
Overall complications			**<0.001**			**<0.001**			**<0.001**
Major surgical complications	6.294	3.625–10.928	**<0.001**	4.637	2.401–8.956	**<0.001**	37.157	7.717–178.926	**<0.001**
Anastomotic leak	12.513	6.531–23.971	**<0.001**	8.647	3.783–19.763	**<0.001**	63.795	15.141–268.784	**<0.001**
Age			**0.020**			0.185			**0.001**
Gender	1.197	0.720–1.992	0.516	1.351	0.769–2.374	0.314	0.689	0.198–2.397	0.760
Type of gastrectomy	1.586	0.936–2.687	0.092	1.443	0.799–2.603	0.247	1.594	0.477–5.328	0.541
Multivisceral resection	0.773	0.434–1.240	0.294	0.738	0.414–1.316	0.317	0.745	0.206–2.690	0.775
Tumor location	0.848	0.507–1.418	0.595	0.976	0.542–1.724	1.000	0.404	0.116–1.408	0.214
Laurén classification	1.316	0.772–2.241	0.356	0.969	0.543–1.728	1.000	4.471	0.950–21.031	0.062
pT category			0.116			0.086			0.971
pN category			0.377			0.174			0.729
AJCC/UICC stage			0.122			0.061			0.931

*p* values in bold type indicate statistical significance. OR: odds ratio; CI: confidence interval.

**Table 7:** Variables associated with postoperative mortality in multivariable analysis.

Variable	Overall Cohort (1972–2014)			Early Group (1972–1992)			Late Group (1993–2014)		
	OR	95% CI	*p*	OR	95% CI	*p*	OR	95% CI	*p*
Major surgical complications	2.550	1.034–6.292	**0.042**	2.319	0.862–6.237	**0.096**	9.774	0.712–134.246	**0.088**
Anastomotic leak	5.550	1.987–15.507	**0.001**	4.000	1.197–13.367	**0.024**	20.391	1.561–266.289	**0.021**
Age	1.030	1.005–1.055	**0.017**				1.191	1.060–1.339	**0.033**

*p* values in bold type indicate statistical significance. OR: odds ratio; CI: confidence interval.
